# Ultrasound-Guided CAPS (Crosswise Approach to Popliteal Sciatic) Block: A Novel Technique for Supine Popliteal Fossa Block

**DOI:** 10.7759/cureus.20894

**Published:** 2022-01-03

**Authors:** Tuhin Mistry, Kartik Sonawane, Vinita Keshri, Jagannathan Balavenkatasubramanian, Chelliah Sekar

**Affiliations:** 1 Anaesthesiology and Perioperative Care, Ganga Medical Centre & Hospitals Pvt Ltd., Coimbatore, IND

**Keywords:** popliteal fossa block, caps block, ultrasound, postoperative pain, acute pain management, regional anaesthesia, popliteal sciatic nerve block

## Abstract

The sciatic nerve block in the popliteal fossa is a popular lower extremity block for below-knee surgeries. Here the sciatic nerve is targated at or just above the point of its divergence into the tibial and common peroneal nerves. Amongst the described techniques, the supine approach of popliteal fossa block offers greatest patient comfort but has a few challenges accessing the nerve. We describe a novel ultrasound-guided distal transverse or crosswise approach to popliteal sciatic (CAPS) block performed in five patients in the supine position without unsteadiness of the knee or hip joint.

## Introduction

The popliteal sciatic nerve block (PSNB) is a widely practiced regional anesthesia (RA) technique to provide anesthesia or analgesia for below-knee surgeries. It is either used alone or in combination with a femoral or saphenous nerve block. The use of ultrasound for PSNB improves the success rate and reduces the risk of complications [1]. The ultrasound-guided PSNB can be performed in the supine, prone, or lateral position [2-4]. The prone or lateral position is convenient for the anesthesiologist but may not always be feasible as it is relatively time-consuming and requires assistance to change the patient’s position. Positional change is also onerous for patients with an associated spine injury, morbid obesity, hemodynamic instability, and pregnancy, as well as those on mechanical ventilation [5]. Hence, PSNB in the supine position has been suggested to provide greater patient comfort. The literature has described various supine PSNB approaches such as posterior, lateral, and medial approaches [6-8]. However, in all the aforementioned approaches, lifting the leg or flexion at hip and knee joints is required to perform the block. Consequently, limb movement results in pain in the non-anesthetized leg or misalignment of non-stabilized fracture segments. Hence, we need a technique to perform the supine PSNB without disturbing the limb position.

The sciatic nerve (SCN) enters the popliteal fossa through its apex and diverges into the tibial nerve (TN) and common peroneal nerve (CPN). However, this divergence can occur at any level from its origin (sacral plexus) to its termination in the popliteal fossa. Cadaveric studies reported the divergence of the SCN at 0-11.5 cm, even up to 18.5 cm above the popliteal crease, where TN and CPN leave the common paraneural sheath [9,10]. Based on this information, one of the authors (T.M.) developed a novel supine lateral approach to ultrasound-guided popliteal fossa block, which we describe as the “crosswise approach to popliteal sciatic block or CAPS block”, as an alternative to the various techniques described in the literature. Here, crosswise means across or transverse. The ultrasound scan along the lateral aspect of the distal thigh aligns the ultrasound beam perpendicular to the SCN, generating a transverse cross-sectional image. This distal transverse approach visualizes the SCN in the short axis medial to the biceps femoris muscle. In this case series, we performed ultrasound-guided CAPS blocks in five random patients of different age groups undergoing below-knee surgeries. We aimed to assess the feasibility of CAPS block as an alternative technique to the described supine approaches.

## Case presentation

For this case series, five patients admitted to our hospital for unilateral below-knee surgeries under the central neuraxial block (CNB) were enrolled. The written consent for the procedure, CAPS block, and publication was obtained from each patient or their relatives. Approval from our Institutional Review Board was also obtained for this case series. The CAPS block was performed in the postanesthesia care unit on the operated limb when full motor power had returned following CNB to the non-operated lower limb (i.e. zero scores on Bromage scale). The demographic parameters, diagnosis, types of surgery, and anesthetic techniques are shown in Table 1.

**Table 1:** Demographic profile ASA-PS, American Society of Anesthesiologists Physical Status; CSEA, combined spinal-epidural anesthesia; CTEV, congenital talipes equinovarus; F, female; M, male; ORIF, open reduction internal fixation; SAB, subarachnoid block

Case No.	Age (years)	Sex (M/F)	Weight (kg)	Height (cm)	ASA-PS	Diagnosis	Surgery	Anesthesia Technique
1	60	M	67	172	II	Closed right distal tibia and fibula fractures	ORIF with plating of tibia and fibula	CSEA
2	47	M	63	165	I	Open left calcaneum fracture	Debridement and pinning	SAB
3	70	F	58	168	II	Closed left trimalleolar ankle fracture and subluxation	ORIF with tubular plating lateral malleolus, medial malleolus TBW, and posterior malleolus cancellous screw fixation	CSEA
4	14	M	45	158	I	Right-sided recurrent right CTEV	Ilizarov fixation	CSEA
5	10	F	35	145	I	Left-sided post-traumatic limb length discrepancy with ipsilateral ankle varus deformity	Corrective osteotomy with lengthening of left tibia with Ilizarov fixation	CSEA

**Figure 1:**
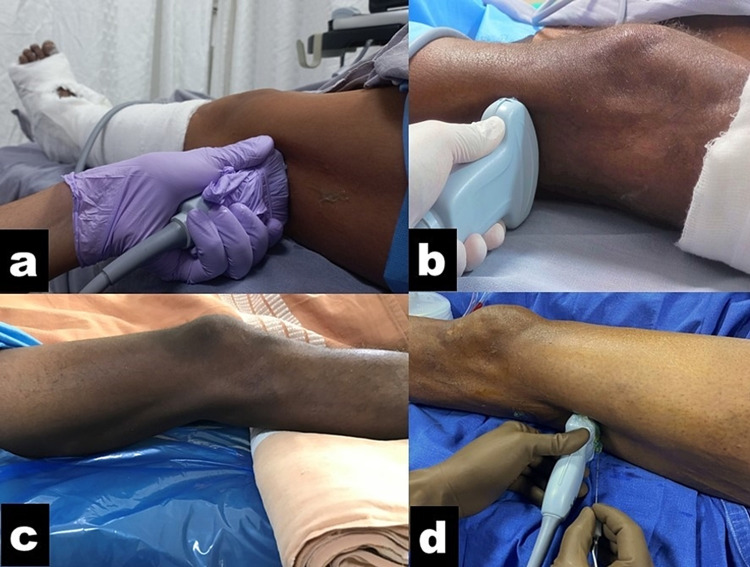
Patient and ultrasound transducer position for the CAPS block (a) Limb position (without any assistance) and placement of a linear probe just above the popliteal crease. (b) Scanning with the curvilinear probe proximal to the popliteal crease. (c) A folded sheet may be placed underneath the calf muscles to create more space for probe placement and needling (optional). (d) Out-of-plane needle insertion for CAPS block at or just above the point of divergence of SCN. CAPS, crosswise approach to popliteal sciatic; SCN, sciatic nerve

The success of the CAPS block technique depends on the appropriate positioning of the ultrasound transducer over the lateral aspect of the distal thigh in the transverse plane and targeting SCN at or above the point of divergence. Hence, CAPS block can be performed at 5-10 cm proximal to the popliteal crease at or above the point of divergence where popliteal vessels may or may not appear in the ultrasound image. A high-frequency linear (Sonosite Edge II HFL 38xp/13-6 MHz, Fujifilm SonoSite Inc., Bothell, WA, USA) or a low-frequency curvilinear (Sonosite rC60xi/5-2 MHz) ultrasound transducer was placed in a transverse plane perpendicular to the skin over the lateral aspect of the lower thigh. It is positioned on or below the intermuscular groove formed by the vastus lateralis and biceps femoris muscles. It corresponds to the upper border of the patella, just proximal to the level of the popliteal crease (Figures 1a, 1b). The linear transducer was used in pediatric and thin-built adult patients.

**Figure 2:**
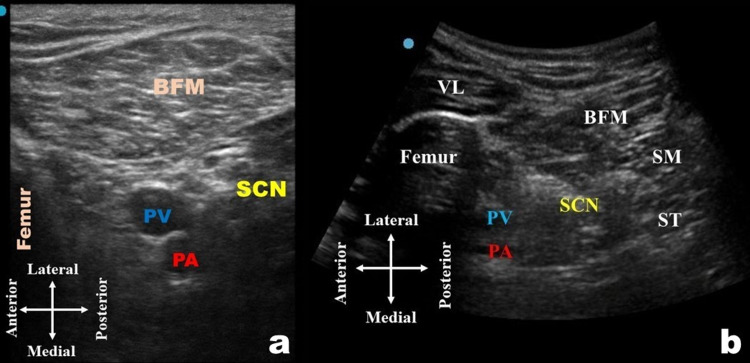
Ultrasound image of the transverse (crosswise) view of the popliteal sciatic nerve (a) Linear transducer. (b) Curvilinear transducer. BFM, biceps femoris muscle; PA, popliteal artery; PV, popliteal vein; SCN, sciatic nerve; SM, semimembranosus muscle; ST, semitendinosus muscle; VL, vastus lateralis muscle

If required, a folded towel, soft pillow, or foam leg elevator may be placed underneath the calf muscles to create room for the probe placement (Figure 1c). The probe was moved in a cephalad direction (Figure 1d) to identify the hyperechoic SCN before the divergence, surrounded by hypoechoic muscles (vastus lateralis, long head of biceps femoris, semimembranosus, and semitendinosus), and the hyperechoic shaft of the femur (Figures 2a, 2b).

**Figure 3:**
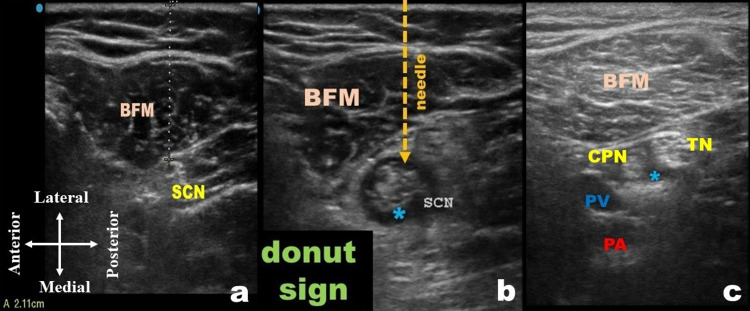
CAPS block (a) Depth of SCN from the skin. (b) Out-of-plane needle placement and subparaneural injection of LA at or just above the divergence of SCN. (c) LA spread around the TN and CPN below the divergence. BFM, biceps femoris muscle; CAPS, crosswise approach to popliteal sciatic; CPN, common peroneal nerve; PA, popliteal artery; PV, popliteal vein; blue asterisk, LA, local anesthetic; SCN, sciatic nerve; TN, tibial nerve Blue asterisk shows LA

The distance of the probe from the popliteal crease, the depth of SCN from the skin on the ultrasound image (Figure 3a), the distinct demarcation of the paraneural sheath, and the subparaneural space were noted. Popliteal vessels may or may not be visible depending on the scanning area. A quantitative score (SCN visibility score) was also recorded, as described by Gürkan et al. [11], where I: SCN is identified, but borders are unclear; II: SCN and borders are delineated, less than or equal to three fascicles are visible; III: SCN and borders are distinguished, more than or equal to four fascicles are visible. The score was used to rank how well the nerve was visualized in the CAPS approach.

A 22-gauge, 100-mm echogenic nerve block needle was inserted perpendicular to the skin from a lateral to medial direction using an out-of-plane technique (Figure 3b). After placing the needle tip in the subparaneural space at or above the divergence and negative aspiration, the LA solution was injected in 3-5 mL increments creating a “donut sign” (Figure 3b) due to circumferential spread around the nerve(s). Upon tracing the SCN distally, the LA spread around the TN, and CPN was visualized to verify the correct anatomic placement of the block (Figure 3c). The block was performed with 20 mL of 0.2% ropivacaine and 4 mg of dexamethasone in three patients; 15 mL of 0.1% ropivacaine and 2 mg of dexamethasone were used in the remaining two cases.

The type of transducer, electrostimulation, number of injections (single or multiple) needed to achieve the “donut sign,” post-block distal scanning to visualize LA spread around two divisions (TN and CPN), and the duration of analgesia was also recorded (Table 2). Postoperatively, all patients received intravenous paracetamol 15 mg.kg^-1^ every six hours and ketorolac 0.5 mg.kg^-1^ every eight hours as a part of multimodal analgesia. The rescue analgesic fentanyl 1 mcg.kg^-1^ was administered when the pain score was 4 on a numeric rating scale, and the time was noted (duration of analgesia). All the patients were followed up for the next 24 hours.

**Table 2:** Block characteristics EMR, evoked motor response; LA, local anesthetic

Case No.	1	2	3	4	5
Transducer used	Curvilinear	Curvilinear	Linear	Linear	Linear
Observed EMR	Plantar flexion, inversion	Dorsiflexion	Plantar flexion, inversion	Plantar flexion, inversion	Dorsiflexion, eversion
Distance of probe from popliteal crease (cm)	5	5.5	6	4	2
Depth of Sciatic nerve from skin (cm)	3.33	2.92	2.11	1.91	1.72
Popliteal vessels seen	Yes	Yes	Yes	Yes	Yes
Paraneural sheath and subparaneural space visible	Yes	Yes	Yes	Yes	Yes
Sciatic nerve visibility score	II	II	III	III	III
No of injection needed to achieve the “donut sign”	Single	Single	Single	Single	Single
LA concentration and volume (ropivacaine + dexamethasone)	0.2% 20 mL + 4 mg	0.2% 20 mL + 4 mg	0.2% 20 mL + 4 mg	0.1% 15 mL + 2 mg	0.1% 15 mL + 2 mg
Post-block LA around both divisions visible	Yes	Yes	Yes	Yes	Yes
Duration of analgesia (hours)	16	18	16	17	18

We used electrostimulation in all the cases to confirm the identity of the SCN and recorded the first evoked motor responses (Table 2). The block was performed within 6 cm proximal to the popliteal crease at or just above the point of divergence of SCN. A single injection inside the paraneural sheath was sufficient to deposit the LA around the SCN. Immediate confirmation of block success was not possible because of the residual effects of CNB. The assessment of the complete return of sensory and motor power was also tricky because of a back-slab plaster. However, none of the patients had pain (static or dynamic) in the early postoperative period, indicating a successful analgesic block. We did not notice block failure or any complications associated with CAPS block.

## Discussion

Our CAPS block technique successfully blocked the SCN in all five patients without technical difficulty or complications. None of the patients needed any human assistance for limb positioning during the performance of the block. The folded sheet was also not required in any of our cases. It provided adequate postoperative analgesia. The SCN, paraneural sheath, and subparaneural space were distinctly visualized. On neurostimulation, plantar flexion and inversion were elicited in three cases and dorsiflexion in two patients.

**Figure 4:**
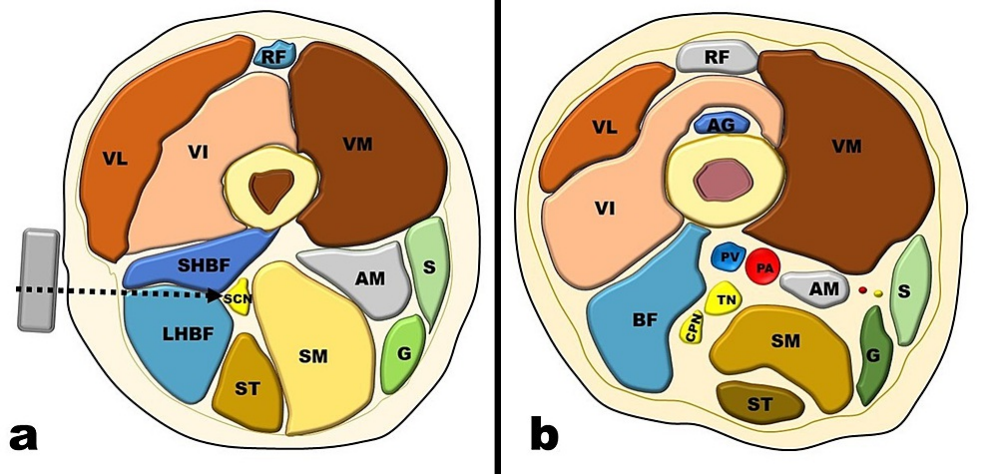
Transverse section of the lower thigh at the popliteal fossa Sciatic Nerve (SCN): (a) Before divergence. (b) After divergence AG, articularis genus muscle; AM, adductor magnus muscle; CPN, common peroneal nerve; G, gracilis muscle; LHBF, long head of the biceps femoris muscle; PA, popliteal artery; PV, popliteal vein; RF, rectus femoris; S, sartorius muscle; SHBF, short head of the biceps femoris muscle; SM, semimembranosus muscle; ST, semitendinosus muscle; TN, tibial nerve; VI, vastus intermedius muscle; VL, vastus lateralis muscle; VM, vastus medialis muscle Gray rectangle shows the transducer, and dashed arrow shows the needle trajectory in the out-of-plane CAPS block

In the popliteal region, the SCN is surrounded laterally by the biceps femoris (long and short heads) and medially by the semitendinosus and semimembranosus muscles (Figure 4a). SCN diverges into the TN and CPN at a variable distance cranial to the popliteal crease (Figure 4b).

The success of the CAPS block technique depends on the appropriate positioning of the ultrasound transducer over the lateral aspect of the distal thigh in the transverse plane and targeting SCN at or above the point of divergence. We observed divergence of SCN about 2 - 6 cm proximal to the popliteal crease while performing CAPS block. However, this divergence can occur at any level from its origin (sacral plexus) to its termination in the popliteal fossa. The SCN divides about 0-11.5 cm cephalad to the popliteal crease, and the superficial femoral artery enters the popliteal fossa as the popliteal artery about 8.5-11.5 cm above the popliteal crease [9,10,12,13]. Hence, CAPS block can be performed at 5-10 cm proximal to the popliteal crease at or above the point of divergence where popliteal vessels may or may not appear in the ultrasound image. The relative position of the popliteal artery and vein also may vary depending on the scanning level.

Sinha and Chan first described the ultrasound-guided PSNB in 2004 in the prone position [2]. The ultrasound scanning of the popliteal fossa and the block can be easily performed in a prone position due to the relatively superficial location of the SCN in this area. However, turning into a prone and then supine position is time-consuming and requires cooperation and effort of the patient, and there may be a risk of airway obstruction in sedated patients [4]. Lateral position can resolve these difficulties to some extent; however, probe stabilization or correct image acquisition can be challenging. Hence, Gray et al. suggested a lateral approach of ultrasound-guided PSNB in the supine position to solve these issues [3]. In 2007, Khabiri et al. described the “gapped supine” position for ultrasound-guided lateral PSNB [4]. Subsequently, supine posterior and supine medial approaches were also introduced [6,7]. The CAPS block differs from previously described supine techniques in the literature as it does not require additional assistance, flexion at the hip and knee joints, or any positioning device.

We believe the CAPS block would overcome the difficulties faced by other techniques. The supine position is convenient for the patient and the anesthesiologist. In the out-of-plane technique, the needle enters perpendicular to the skin. A shorter needle trajectory can cause less tissue damage than in-plane needling. Hence, it would be less painful for awake and unanesthetized patients. The chance of vascular puncture or LA systemic toxicity is also relatively less as it is performed away from vessels. This is a case series that has its limitations. The performer's experience might play a role in the success. The motor block effect of the CAPS block needs to be evaluated especially in the light of enhanced recovery after surgery. Also, the feasibility of CAPS block in obese patients and catheter placement for continuous CAPS block needs to be evaluated. Thus, the CAPS block requires further investigation in the form of prospective randomized controlled studies, cadaver studies, and dye studies in both pediatric and adult age groups.

## Conclusions

CAPS block is an easy, safe, and effective alternative approach to PSNB. We performed the CAPS block without altering the limb position or experiencing technical difficulties. It successfully provided the desired analgesia in all patients. We believe that the CAPS approach is simple and convenient for the patient and the anesthesiologist. Combining CAPS block with saphenous nerve block can provide complete analgesia for below-knee surgeries. However, further research and comparative trials are needed to evaluate its analgesic efficacy and safety profile over other approaches.
